# Glutathione peroxidase 4 restrains temporomandibular joint osteoarthritis progression by inhibiting ferroptosis

**DOI:** 10.1111/jcmm.18377

**Published:** 2024-04-30

**Authors:** Chunyan Pang, Hongmei Zhang, Yi Liu, Na Tang, Kun Tian, Yandong Mu, Xue Li, Li Xiao

**Affiliations:** ^1^ Department of Stomatology, Sichuan Provincial People's Hospital University of Electronic Science and Technology of China Chengdu Sichuan China; ^2^ Department of Laboratory Medicine, Sichuan Provincial Key Laboratory for Human Disease Gene Study, Center for Medical Genetics Sichuan Academy of Medical Sciences & Sichuan Provincial People's Hospital Chengdu Sichuan China

**Keywords:** ferroptosis, fibroblast‐like synoviocytes, GPX4, TMJOA

## Abstract

There are few effective therapeutic strategies for temporomandibular joint osteoarthritis (TMJOA) due to the unclear pathology and mechanisms. We aimed to confirm the roles of GPX4 and ferroptosis in TMJOA progression. ELISA assay was hired to evaluate concentrations of ferroptosis‐related markers. The qRT‐PCR assay was hired to assess gene mRNA level. Western blot assay and immunohistochemistry were hired to verify the protein level. CCK‐8 assay was hired to detect cell viability. Human fibroblast‐like synoviocytes (FLSs) were cultured to confirm the effects of GPX4 and indicated inhibitors, and further verified the effects of GPX4 and ferroptosis inhibitors in TMJOA model rats. Markers of ferroptosis including 8‐hidroxy‐2‐deoxyguanosine (8‐OHdG) and iron were notably increased in TMJOA tissues and primary OA‐FLSs. However, the activity of the antioxidant system including the glutathione peroxidase activity, glutathione (GSH) contents, and glutathione/oxidized glutathione (GSH/GSSG) ratio was notably inhibited in TMJOA tissues, and the primary OA‐FLSs. Furthermore, the glutathione peroxidase 4 (GPX4) expression was down‐regulated in TMJOA tissues and primary OA‐FLSs. Animal and cell experiments have shown that ferroptosis inhibitors notably inhibited ferroptosis and promoted HLS survival as well as up‐regulated GPX4 expression. Also, GPX4 knockdown promoted ferroptosis and GPX4 overexpression inhibited ferroptosis. GPX4 also positively regulated cell survival which was the opposite with ferroptosis. In conclusion, GPX4 and ferroptosis regulated the progression of TMJOA. Targeting ferroptosis might be an effective therapeutic strategy for TMJOA patients in the clinic.

## 1 INTRODUCTION

Temporomandibular disorders (TMD) are characterized by high morbidity and complicated.^1^ Patients with temporomandibular joint osteoarthritis (TMJOA) have a low quality of life and suffer from severe pain and difficulty swelling.^2^ However, there are few effective therapeutic strategies for TMJOA due to the unclear pathology and mechanism for its progression. Thus, it is urgent to reveal the pathology and mechanism of TMJOA progression to develop an effective strategy in the clinic.

Cartilage degradation, bone homeostasis dysfunction, and chronic inflammation could be observed in the TMJOA microenvironment.^3^ Therefore, all factors that were involved in bone homeostasis and inflammation would influence the progression of TMJOA. NF‐κB was a well‐known pathway that promoted inflammation.^4^, ^5^ It was reported that the antioxidant resveratrol retained cell apoptosis and TMJOA progression by targeting the NF‐κB signalling pathway.^6^ Yohimbine inhibited inflammation and bone damage by suppressing the NF‐κB signalling pathway.^7^ Yohimbine also decreased the production of cytokines of inflammatory cells as well as prevented apoptosis.^7^ Other factors including ROS, and lipid peroxidation might also be associated with inflammation and TMJOA.^8^

Ferroptosis was first named in 2012 and was an iron‐dependent biological process that controlled cell death.^9^ Accumulating evidence proved that ferroptosis was closely related to inflammation, ROS, and lipid peroxidation.^10^ Also, the ferroptosis of chondrocytes could promote osteoarthritis progression by increasing the production of several cytokines and activating the Nrf2 signalling.^11^ Targeting ferroptosis by D‐mannose also inhibited osteoarthritis progression.^12^ It was reported that ferroptosis was negatively regulated by Glutathione peroxidase 4 (GPX4).^13^ Mechanically, GPX4 employed GSH to inhibit lipid peroxidation and suppress ferroptosis.^14^ Due to the role of ferroptosis in inflammation and osteoarthritis, GPX4 was also involved in osteoarthritis.^15^, ^16^ However, there was no evidence to prove the role of ferroptosis and GPX4 in TMJOA pathology and mechanisms.

Herein, our study uncovered that GPX4 and ferroptosis could regulate the progression of TMJOA. GPX4 overexpression notably promoted cell survival of HLS by inhibiting ferroptosis. Inhibiting ferroptosis with ferroptosis inhibitors notably protected HLS cells. Targeting ferroptosis might be an effective therapeutic strategy for TMJOA patients in the clinic.

## 2 MATERIALS AND METHODS

### 2.1 Patients and specimens

Synovial samples were acquired from 16 patients with TMJOA who underwent arthroplasty surgery and six normal patients. The normal patients were patients with temporomandibular fractures. TMJOA was excluded by verbally asking if there were symptoms such as pain before the fracture. Patients were included based on the diagnostic criteria for TMD.^17^ Exclusion criteria included a history of joint trauma, previous treatment of TMD and the presence of systemic joint disease. Specimens were divided into damaged areas (designed as OA) and normal areas. All human studies were approved by the ethics committee of Sichuan Provincial People's Hospital (Approval Number: Lunshen (Yan) 2022‐233). Before the operative procedure, all patients and volunteers had signed the full written consent. All procedures were carried out according to the Declaration of Helsinki.

### 2.2 Synovial fluid collection

Synovial fluid samples were harvested during TMJ arthrocentesis. the upper chamber of the TMJ cavity was injected with 1.0 mL of saline, mixed with synovial fluid three times, and repeatedly aspirated before reinjection. Then the synovial fluid (1.0 mL) was collected, and the samples with blood were discarded. After centrifugation (1500 g for 10 min at 4°C), the samples were packed and stored at −80°C.

### 2.3 Collection of TMJ tissues and synovial tissues

After the patient was under general anaesthesia, a small incision was made in the para‐auricular area, arthroscopy and other surgical instruments were inserted, intra‐articular conditions were observed, and human TMJ synovial tissue and TMJ tissue were collected.

### 2.4 Patient sample workflow

TMJOA synovial fluid was applied in an Enzyme‐linked immunosorbent assay (ELISA) assay to measure 8‐OHdG content. TMJ tissues were applied for haematoxylin–eosin (H&E), safranin O/fast green, and alcian blue staining to determine cartilage tissue damage. Synovial fluid was applied in immunohistochemistry (IHC), qRT‐PCR, western blot, Iron assay, and ELISA experiments to determine ferroptosis.

### 2.5 Primary HLSs isolate and culture

Primary HLSs were isolated from synovial tissues. Briefly, synovial tissues were divided into pieces and digested with 0.25% trypsin at 37°C for 30 min. Then tissues were washed with cold PBS three times followed by digestion using 0.25% collagenase II at 37°C for 8 h. The cell suspension was filtered using a 70 mm cell strainer followed by a centrifuge (1000 rpm) for 5 min to obtain primary synoviocytes. Cells were seeded in a completed culture medium. After three passages, synoviocytes (N‐FLSs and OA‐FLSs) were used for Iron, ELISA (Glutathione peroxidase activity, GSH content, GSH/GSSG, and MDA), qRT‐PCR (GPX4), and western blot (GPX4, ADAMTS5, SLC3A2, ACSL4) assays.

### 2.6 Cell culture

Human fibroblast‐like synoviocytes (HLS) were purchased from the Shanghai Institute of Biochemistry and Cell Biology, Chinese Academy of Sciences (Shanghai, China). Cells were cultured in DMEM supplemented with 10% fetal bovine serum at 37°C mixed with a humidified atmosphere with 5% CO_2_.

### 2.7 Plasmid's transfection

GPX4 knockdown plasmids and overexpression plasmids were obtained from Xiayangxin (Chengdu, China). Plasmids were transfected into the purchased human HLSs using Lipofectamine 2000 (Life Technologies) as the instructions indicated. After 72 h of transfection, cells were treated with 2 mg/mL puromycin (Gibco, California, USA) for several days. Then the treated HFLSs were applied to assess the effects of GPX4 and ferroptosis using CCK‐8, ELISA, western blot, qRT‐PCR and Iron assays.

### 2.8 ELISA assay

ELISA assay was employed to detect concentrations of 8‐OHdG in TMJOA synovial fluid. The levels of Glutathione peroxidase, GSH/GSSG and MDA in the supernatant of lysed cells were assessed with a rat 8‐OHdG ELISA kit (ml028318, MLBIO, Shanghai, China), Glutathione peroxidase ELISA kit (S0056, Beyotime Biotechnology, Shanghai, China), GSH/GSSG ELISA kit (S0053, Beyotime Biotechnology, Shanghai, China) and MDA ELISA kit (S0131S, Beyotime Biotechnology, Shanghai, China) as instructions indicated.

### 2.9 Haematoxylin–eosin/Safranin O/fast green/Alcian blue staining

Four‐μm‐thick sections of formalin‐fixed paraffin‐embedded synovial tissues were deparaffinized and rehydrated with various concentrations of alcohol. For Haematoxylin–eosin staining, sections were stained with haematoxylin (Beyotime Biotechnology) for 5 min. Sections were then stained with eosin for 2 min (Beyotime Biotechnology) after being infused with hydrochloric acid‐ethanol and immersed in tap water for 15 min. For Safranin O/fast green/Alcian blue stain, slides were then washed with distilled water and incubated in 0.04% Fast Green for 15 min at room temperature. Slides were then washed with distilled water and incubated in 0.1% Fast Green and 0.04% Safranin O or Alcian blue in saturated picric acid for 30 min. Then, they were dehydrated and mounted with DPX Mounting. Images were acquired by the Aperio ScanScope CS2 device (Aperio Technologies, Vista, CA, USA).

### 2.10 Iron assay

Iron Assay Kit (MAK025, Sigma Aldrich) was hired to assess iron concentration. Briefly, 5 × 10^6^ cells were seeded in six‐well plates and then pretreated with Erastin, Fer‐1, or DFO for 24 h. About 1–50 μL samples were added into sample wells and make sure the volume to 100 μL per well with Assay Buffer. Then 5 μL of iron assay buffer was added to each sample. For total iron evaluation, also 5 μL of iron reducer was added into each of the sample wells to reduce Fe^3+^ to Fe^2+^. Next, a horizontal shaker was used to mix the wells and incubated for 30 min at 25°C. Then, each sample was added 100 ul of iron probe and incubated the reaction for 60 min at 25°C. The absorbance was measured at 593 nm by EnSpire Multimode Plate Reader (PerkinElmer, Waltham, MA, USA).

### 2.11 RNA extraction and quantitation

Total RNA samples were extracted from cultured cells or tissues using the Total RNA Extraction Kit (#17200; AmyJet Scientific, Wuhan, China) following the manufacturer's instructions. The Nanodrop 2000 instrument (Thermo Fisher Scientific) was employed to detect the mRNA concentration. As the instruction indicated, about 1 μg total RNA was used to synthesize the cDNA with the Omniscript RT Kit (#205111; Qiagen). Then, as the instructions indicated, the real‐time quantitative PCR assay was performed using the TransStart® Green qPCR SuperMix kit (#AQ101‐01; TransGen Biotech, Beijing, China). The standard 2^−△△Ct^ method was used to assess the expression of mRNAs based on at least three biological replicates. The primer sequences we used are shown below GPX4–forward primer: 5'‐TTGGTCGGCTGGACGAGG‐3', GPX4–reverse primer: 5'‐ATGTGCCCGTCGATGTCCTT‐3'; GAPDH–forward primer: 5'‐TTCTTTTGCGTCGCCAGCC‐3', GAPDH–reverse primer: 5'‐CCGTTCTCAGCCTTGACGGT‐3'.

### 2.12 IHC assay

In brief, the tissues were deparaffinized with xylene and rehydrated with the various concentrations of ethanol; the slides were incubated with 3% (v/v) hydrogen peroxide followed by antigen retrieval in the citrate‐mediated high‐temperature. Then slides would be incubated with the primary antibody GPX4 (1:100, abcam, ab125066) at 4°C overnight secondary antibody for 1 h, and stained with diaminobenzidine (DAB). The images were photographed with a microscope. There are four levels of scoring based on the intensity of cellular staining, with no positive staining (negative) scoring 0 points, pale yellow (weakly positive) scoring 1 point, brownish‐yellow (positive) scoring 2 points and brownish brown (strongly positive) scoring 3 points.

### 2.13 Western blot analysis

The total protein was extracted from tissues or HLSs using radioimmunoprecipitation assay (RIPA) to extract the total protein and the bicinchoninic acid (BCA) (Beyotime, Shanghai, China) method was employed to quantify the concentration. The protein sample was first electrophoresed for 2 h, and then we transferred the total protein onto polyvinylidene difluoride membranes (Millipore, Billerica, MA, USA). TBST containing 5% skim milk was used to block the non‐specific antigens for 1 h following incubating with primary antibodies: GPX4 (Abcam, ab12506, 1: 1000), ADAMTS5 (Abcam, ab41037, 1:800), SLC3A2 (protein tech, Cat No: 15193‐1‐AP, 1:10000), ACSL4 (protein tech, Cat No: 66617‐1‐Ig, 1:5000), GAPDH (protein tech, Cat No: 60004‐1‐Ig, 1:10000) and secondary antibodies. The bands were exposed to enhanced chemiluminescence (ECL) and analysed by Image Software (NIH, Bethesda, MD, USA).

### 2.14 Cell viability

Cell viability was evaluated by Cell Counting Kit‐8 (CCK‐8) assay. About 1500 cells were seeded into 96‐well plates. Cells were pretreated with Erastin or Fer‐1/DFO The absorbance was assessed after 2 days by CCK‐8 (Dojindo, Japan).

### 2.15 TMJOA rat model

A total of 32 Sprague–Dawley (SD) rats (8‐week‐old) were maintained in a controlled SPF environment. This study strictly complied with the ethical guidelines established by the Animal Research Ethics Committee of Sichuan Provincial People's Hospital. The rats were randomly assigned to one of four groups: the Sham group (saline injection, *n* = 8), TMJOA group (MIA injection, *n* = 8), TMJOA + Fer‐1 group (*n* = 8), and TMJOA + GPX‐4 overexpression group (*n* = 8). Subsequently, rats were anaesthetised using a 1.5% isoflurane‐oxygen solution. Following established protocols, a 27‐gauge, 0.5‐inch needle was inserted into the anterior upper aspect of the coronoid process to bilaterally inject the reagent (1 mg of MIA, Sigma, St. Louis, MO, USA, catalogue number I2512, dissolved in 50 μL of saline) into the upper chamber of the TMJ to induce TMJOA. For Fer‐1 treatment, the TMJOA model rats were injected with Fer‐1. After 4 weeks, the rats were euthanized, and TMJ tissue and synovial tissue were collected. The histological and immunohistochemical analyses were conducted using TMJ tissues. TMJ synovial tissues were applied for Iron and ELISA assays.

### 2.16 Histological examination

At the end of the intervention, the rats were anaesthetised with 1% sodium pentobarbital (40 mg/kg) and sacrificed, and the TMJ tissues were harvested. The TMJ tissues from each rat group were meticulously dissected, fixed overnight in 4% paraformaldehyde, dehydrated, decalcified in ethylenediaminetetraacetic acid and paraffin‐embedded. Subsequently, the samples were sectioned into 5 μm slices and stained with Fast Green and Safranin O, with joint pathology being further quantified using the OARSI scoring system.

### 2.17 Behavioural testing

Pain behaviour was evaluated by determining the head withdrawal threshold (HWT) using an automated electronic von Frey device (IITC, California, USA). Before HWT measurements, rats were allowed a 1‐week adaptation period to the testing environment. During HWT measurements, rat head movement was not constrained, although mobility was restricted. Von Frey filaments were employed to assess TMJ mechanical sensitivity. A plastic probe was applied to the temporomandibular joint area, specifically the midpoint between the tragus and the outer edge. Positive responses, indicative of pain, included head jerking or shaking. The minimum applied force that induced head withdrawal was recorded as the HWT.

### 2.18 Statistical analysis

All experiments were repeated more than three times and all the data were shown as means ± SD (standard deviation). Statistics and graphs were performed in SPSS 25.0 (SPSS Inc, Chicago, IL) and GraphPad Prism 8.0 (La Jolla, CA, USA). Significant differences between groups were calculated by the *t*‐test. A *p*‐value of <0.05 was considered a significant difference.

## 3 RESULTS

### 3.1 Ferroptosis was activated in TMJOA

**FIGURE 1:**
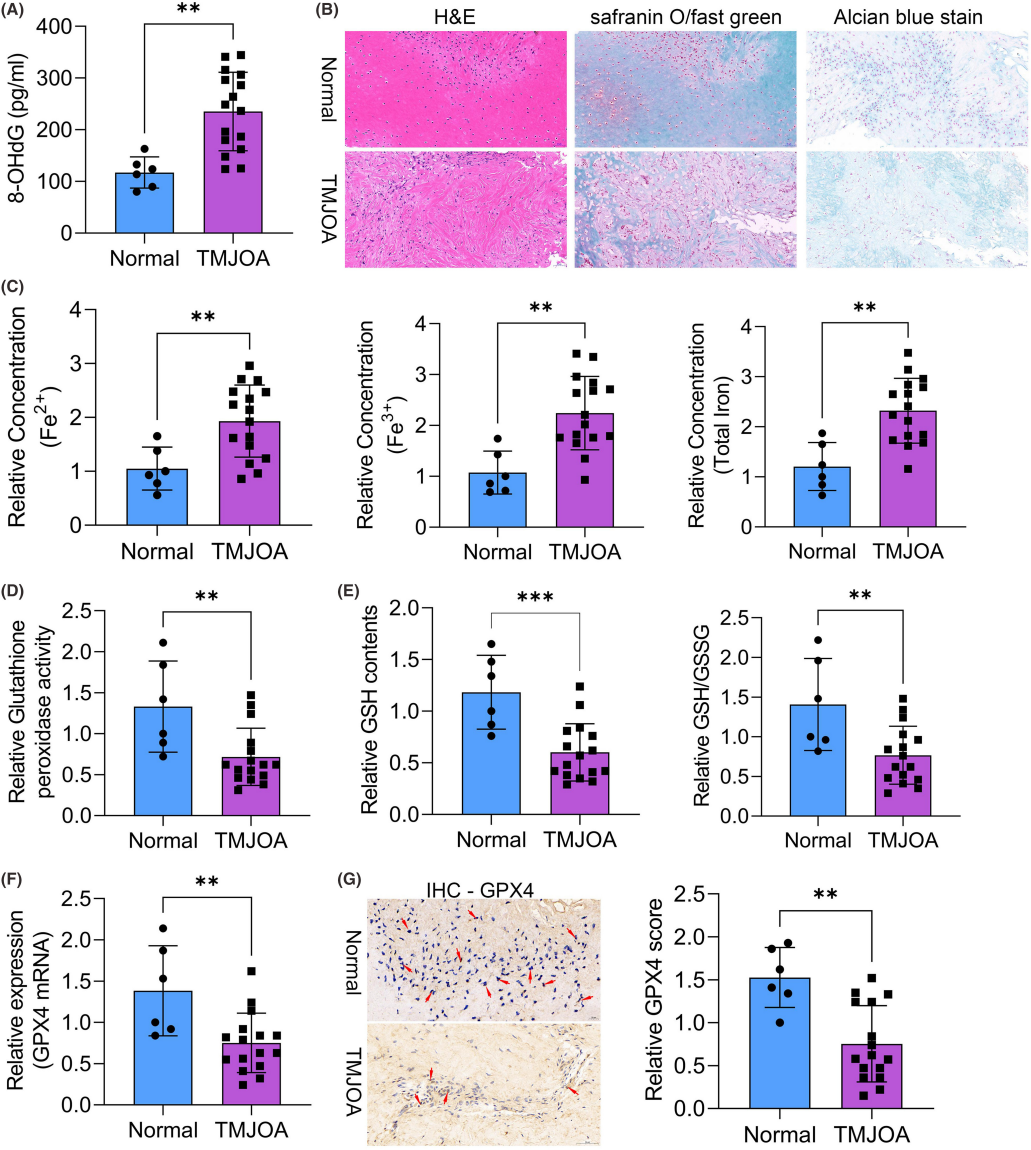
Ferroptosis was activated in TMJOA. (A) 8‐OHdG level in synovial fluid from TMJOA patients and normal patients (patients with temporomandibular fractures). (B) Representative histopathological haematoxylin–eosin, safranin O/fast green and alcian blue staining of undamaged and TMJ tissues from TMJOA patients. (C) Fe^2+^, Fe^3+^, and total iron levels were analysed in synovial tissues from TMJOA and normal patients. (D) Glutathione peroxidase activity levels were detected in synovial tissues from TMJOA and normal patients. (E) GSH contents and ratio of GSH/GSSG were measured in synovial tissues from TMJOA and normal patients. (F) The mRNA level of GPX4 was determined by qRT‐PCR assay in synovial tissues from TMJOA and normal patients. (G) The protein level of GPX4 was determined by western blot assay in synovial tissues from TMJOA and normal patients. **p < 0.01; ***p < 0.001.

To verify the role of ferroptosis in TMJOA progression, we detected several markers in samples from patients and healthy volunteers. A critical marker involved in oxidative stress, 8‐OHdG, was notably increased in TMJOA synovial fluid (Figure 1A). Then we evaluated the cartilage damage using several staining methods. Significant damage was observed in the TMJOA tissues (Figure 1B). The concentrations of different forms of iron were all notably increased in the TMJOA samples (Figure 1C). In contrast, the activity of the antioxidant system including the glutathione peroxidase activity, GSH contents as well as GSH/GSSG ratio was notably inhibited in TMJOA synovial tissues (Figure 1D,E). Furthermore, the expression of a ferroptosis regulator, GPX4, was also down‐regulated in TMJOA synovial tissues compared to the healthy tissues (Figure 1F,G). Overall, we uncovered that ferroptosis was critical for the progression of TMJOA.

### 3.2 Ferroptosis was activated in the primary OA‐FLSs

**FIGURE 2:**
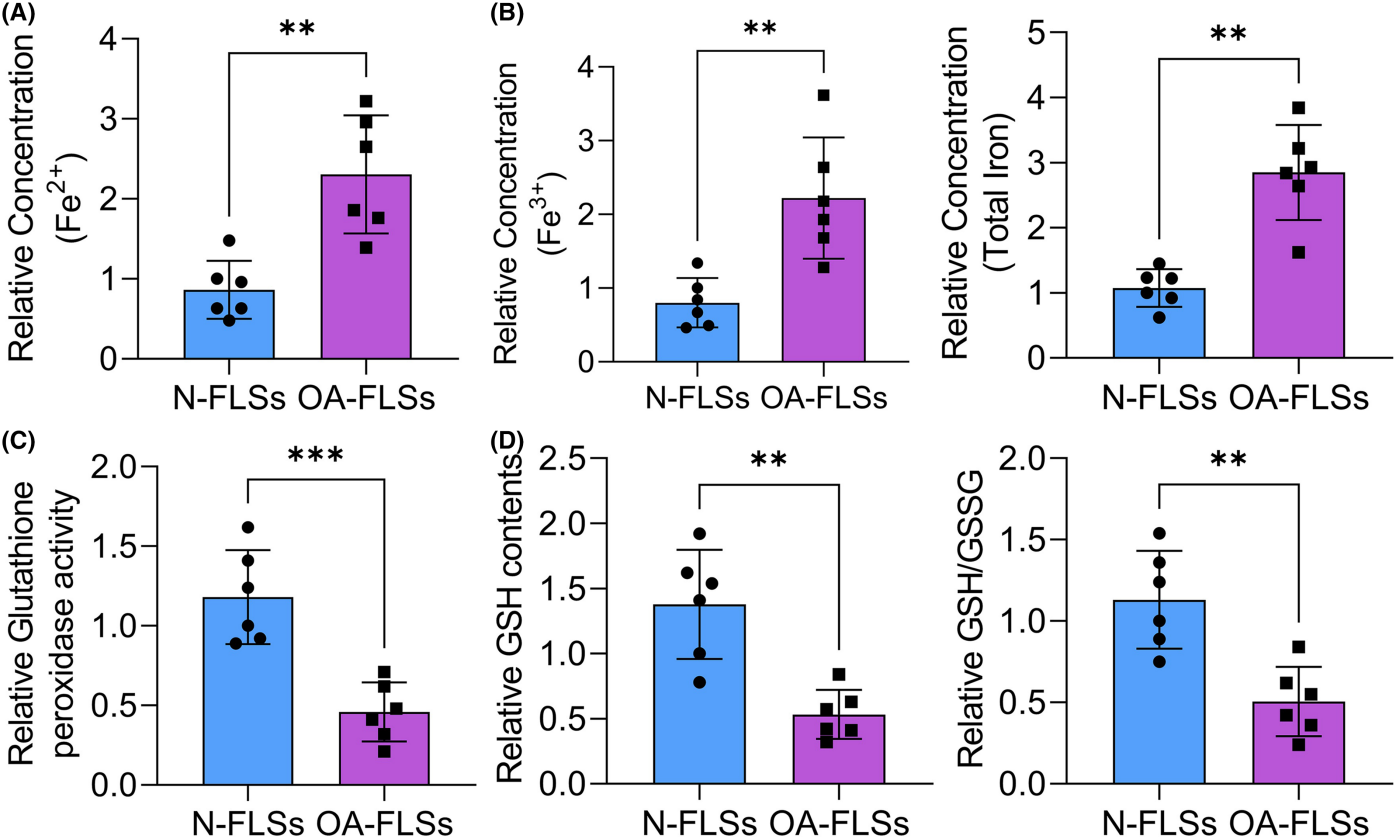
Ferroptosis was activated in the primary OA‐FLSs. (A) Fe^2+^, Fe^3+^, and total iron levels in N‐FLSs and OA‐FLSs. (B) Glutathione peroxidase activity levels were detected in N‐FLSs and OA‐FLSs. (C) GSH contents and ratio of GSH/GSSG were measured in N‐FLSs and OA‐FLSs. **p < 0.01.

**FIGURE 3:**
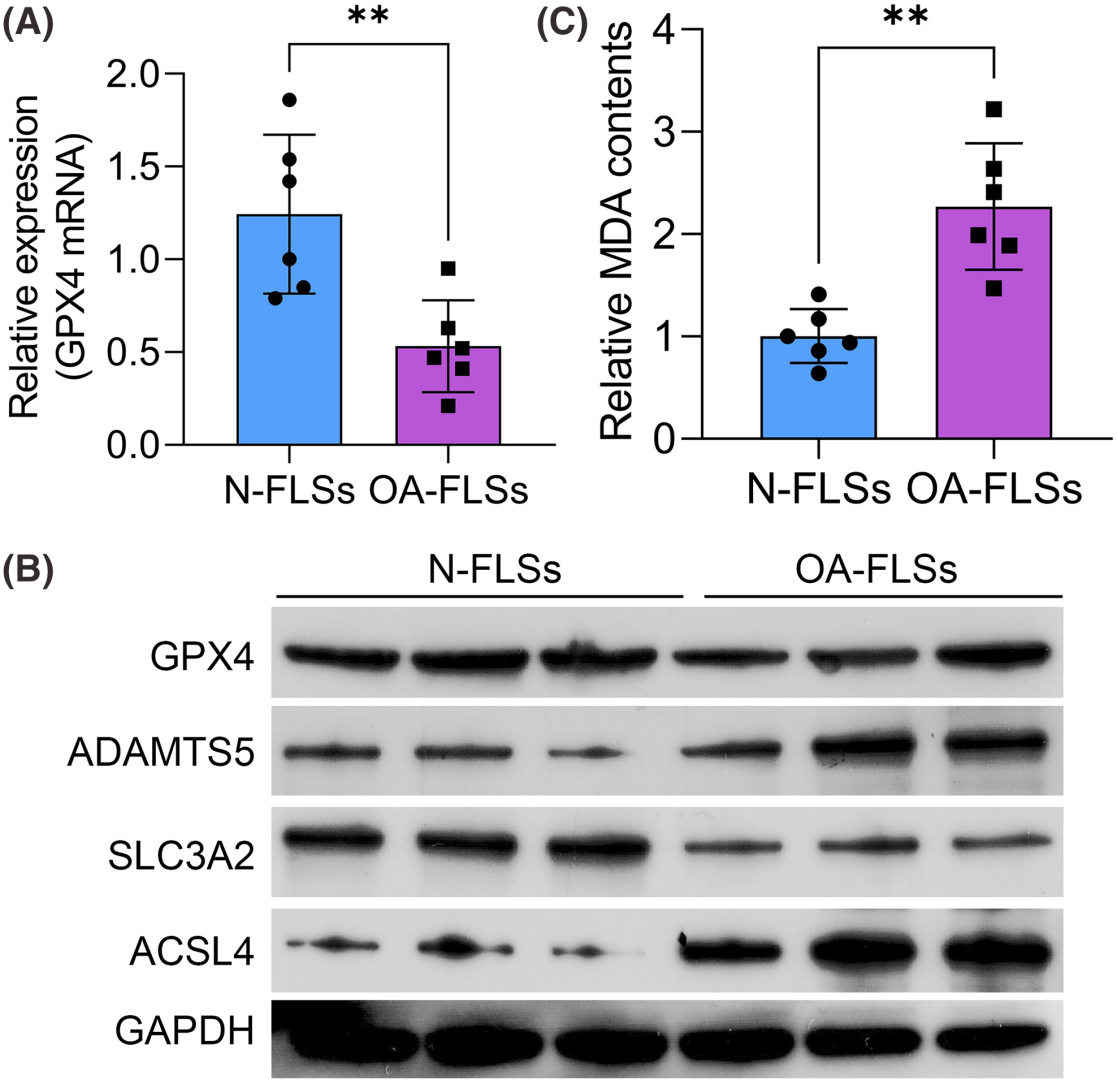
GPX4 was down‐regulated in OA‐FLSs. (A) The mRNA level of GPX4 in N‐FLSs and OA‐FLSs was determined by qRT‐PCR assay. (B) The protein levels of GPX4, ADAMTS5, SLC3A2, ACSL4, and GAPDH in N‐FLSs and OA‐FLSs were determined by western blot assay. **p < 0.01.

Then we isolated and cultured the primary FLSs. The concentrations of different forms of iron were all notably increased in the OA‐FLSs (Figure 2A). In contrast, the activity of the antioxidant system including the glutathione peroxidase activity, GSH contents as well as GSH/GSSG ratio was notably reduced in OA‐FLSs (Figure 2B,C). Furthermore, GPX4 was also down‐regulated in OA‐FLSs compared to the N‐FLSs (Figure 3A,B). Also, SLC3A2 was notably decreased in OA‐FLSs. However, the expression of ADAMTS5 and ACSL4 was notably increased in the OA‐FLSs. The content of MDA was detected with the ELISA assay and notably increased in the OA‐FLSs. These results indicated that lipid peroxidation and ferroptosis were activated in the OA‐FLSs.

### 3.3 Ferroptosis inhibitors promote HFLS survival by suppressing ferroptosis

**FIGURE 4:**
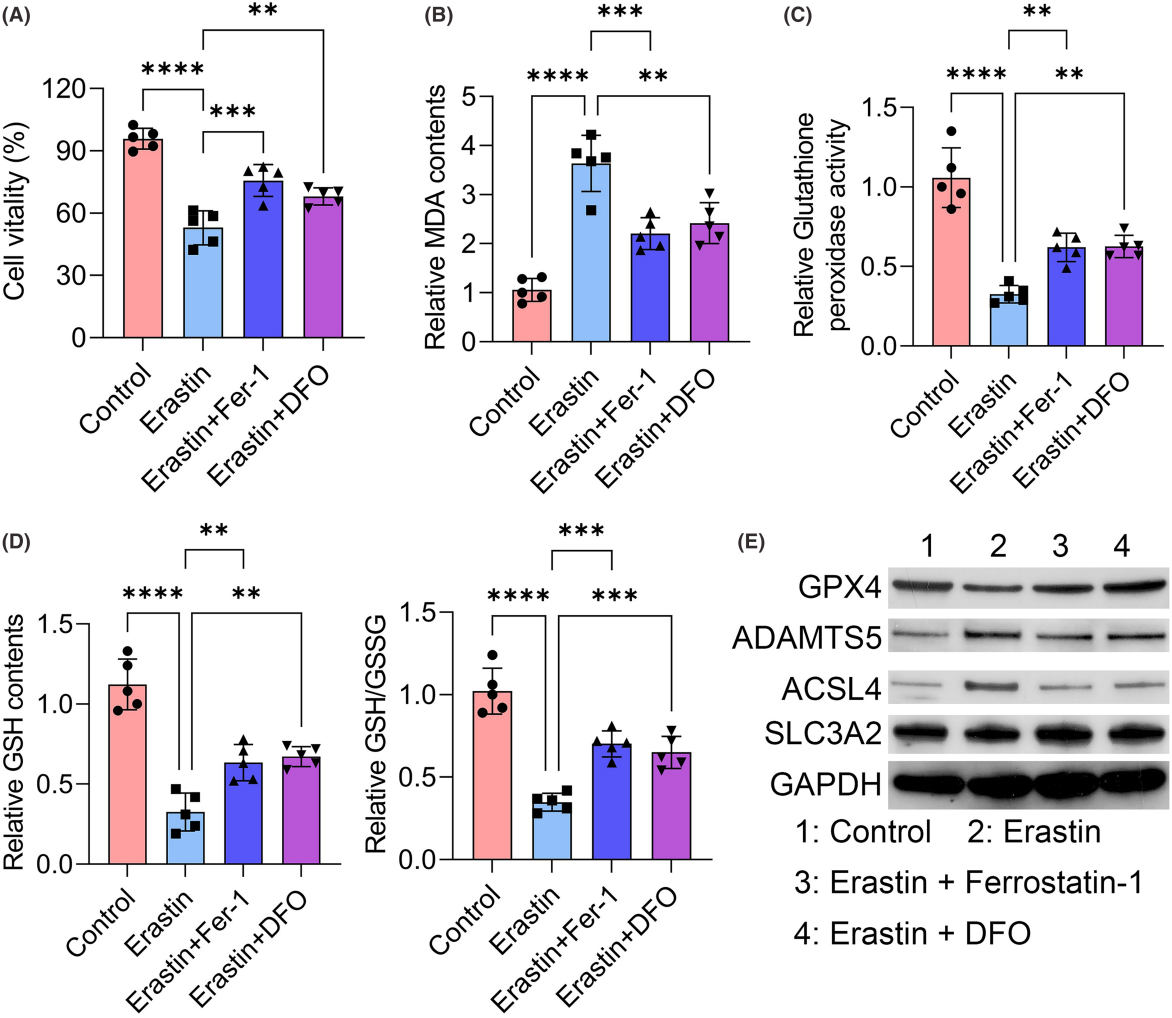
Ferroptosis inhibitors promote HFLSs survival by suppressing ferroptosis. (A) Cell viability was assessed by CCK‐8 assay in indicated groups. (B) The concentration of MDA was assessed by ELISA assay in indicated groups. (C) Glutathione peroxidase activity levels were detected in indicated groups. (D) GSH contents and ratio of GSH/GSSG were measured in indicated groups. (E) The protein levels of GPX4, ADAMTS5, SLC3A2, ACSL4, and GAPDH in indicated groups were determined by western blot assay. **p < 0.01; ***p < 0.001; ****p < 0.0001.

To confirm the correlation between ferroptosis and TMJOA progression, Erastin was used to induce ferroptosis in the HFLSs. Consistent with previous results, Erastin notably inhibited the cell viability of HFLSs. However, the Fer‐1 and DFO treatment rescued the effects of Erastin and promoted cell survival of HFLSs (Figure 4A). To detect the roles of Fer‐1 and DFO in the antioxidant system and ferroptosis, we detected several markers in each group. Erastin treatment increased the content of MDA and Fer‐1 and DFO treatment rescued its effect and reduced the level of MDA of HFLSs (Figure 4B). Also, Erastin treatment decreased glutathione peroxidase activity, GSH contents as well as GSH/GSSG ratio. Fer‐1 and DFO treatment rescued its effect as well as increased the activity of the antioxidant system in HFLSs compared to the Erastin‐treated group (Figure 4C,D). Moreover, GPX4 expression was also down‐regulated in Erastin‐treated HFLS cells compared to the control group (Figure 4E). Also, SLC3A2 was notably decreased in the Erastin‐treated HFLS cells. However, the expression of ADAMTS5 and ACSL4 were notably up‐regulated in the Erastin group (Figure 4E). As shown in Figure 4E, Fer‐1 and DFO treatment notably rescued the role of Erastin in the expression of GPX4, ADAMTS5, ACSL4 and SLC3A2 (Figure 4E). Thus, we proved that ferroptosis inhibitors promoted HFLS survival through suppressing ferroptosis.

### 3.4 GPX4 knockdown inhibited HFLS survival by activating ferroptosis

**FIGURE 5:**
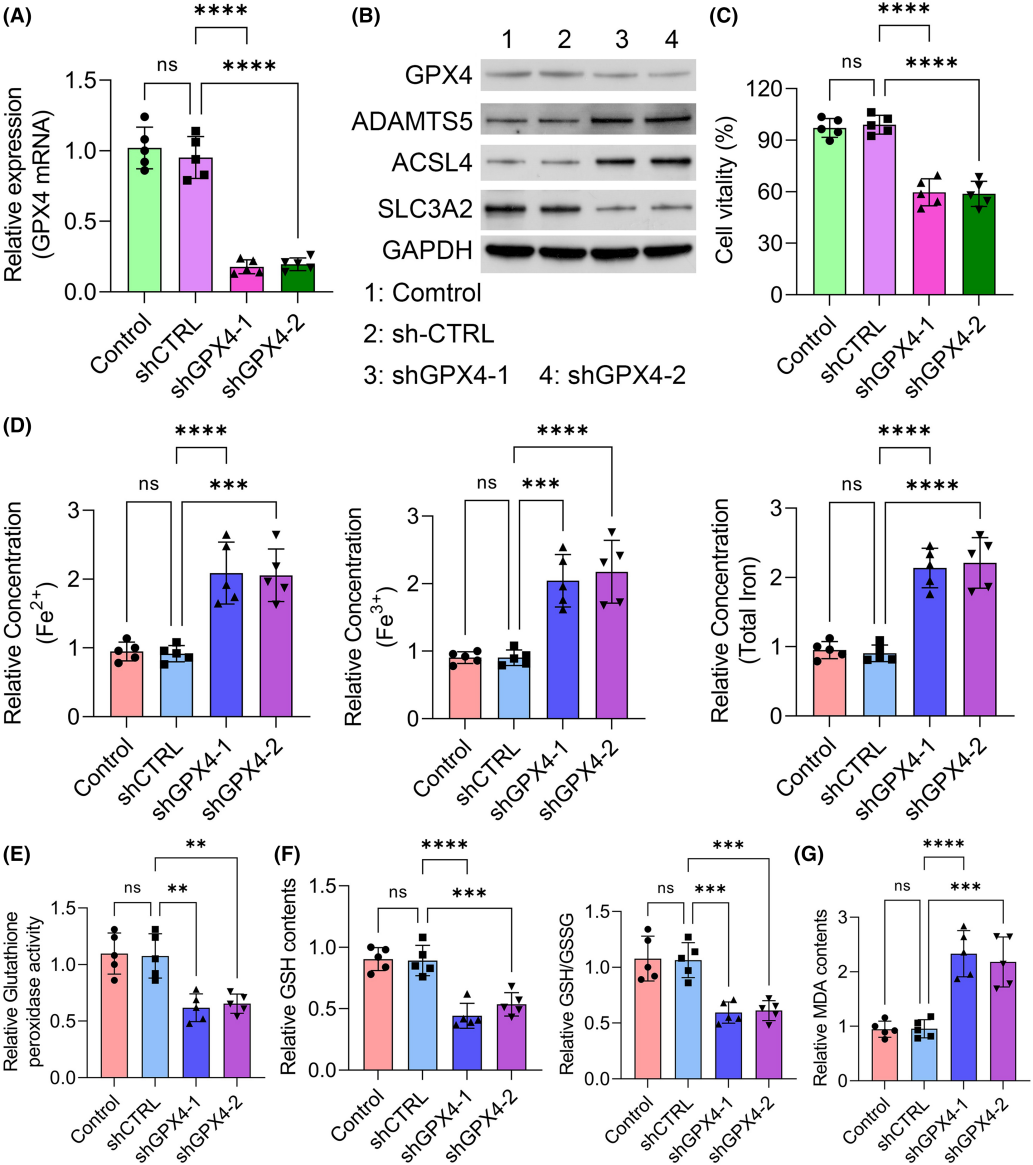
GPX4 knockdown inhibited HFLSs survival by activating ferroptosis. (A) The mRNA level of GPX4 in indicated groups was determined by qRT‐PCR assay. (B) The protein levels of GPX4, ADAMTS5, SLC3A2, ACSL4, and GAPDH in indicated groups were determined by western blot assay. (C) Cell viability was assessed by CCK‐8 assay in indicated groups. (D) Fe^2+^, Fe^3+^, and total iron level in indicated groups. (E) Glutathione peroxidase activity levels were detected in indicated groups. (F) GSH contents and ratio of GSH/GSSG were measured in indicated groups. (G) The concentration of MDA was assessed by ELISA assay in indicated groups. **p < 0.01; ***p < 0.001; ****p < 0.0001.

To confirm the correlation between GPX4 and TMJOA progression, we knocked down GPX4 with two independent shRNAs in the HFLSs. The efficacy of knockdown was confirmed (Figure 5A,B). Furthermore, SLC3A2 was notably decreased in the GPX4 knockdown HFLSs. However, the expression of ADAMTS5 and ACSL4 were notably up‐regulated in the GPX4 knockdown group (Figure 5B). Consistent with previous results, GPX4 knockdown notably inhibited the cell viability of HFLSs (Figure 5C). To evaluate the role of GPX4 in the antioxidant system and ferroptosis, we detected several markers in each group. The concentrations of different forms of iron were all notably increased when GPX4 was knocked down (Figure 5D). In contrast, the activity of the antioxidant system including the glutathione peroxidase activity, GSH contents as well as GSH/GSSG ratio was notably reduced in GPX4 knockdown groups (Figure 5E,F). Moreover, GPX4 knockdown increased the content of MDA of HFLSs (Figure 5G). In conclusion, we found that GPX4 knockdown inhibited HFLS survival through activating ferroptosis.

### 3.5 GPX4 overexpression promoted HFLS survival by inhibiting ferroptosis

**FIGURE 6:**
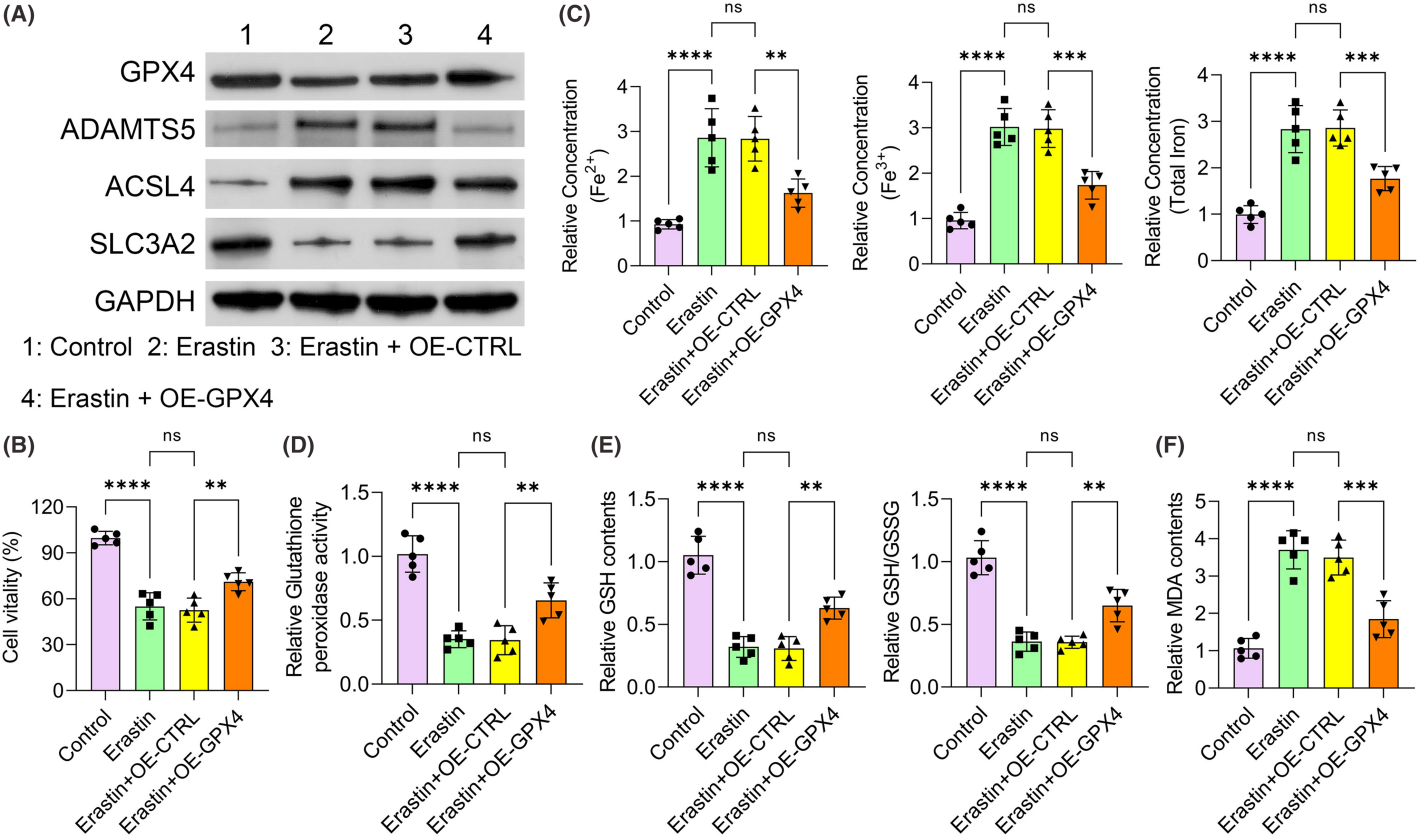
GPX4 overexpression promoted HFLSs survival by inhibiting ferroptosis. (A) The protein levels of GPX4, ADAMTS5, SLC3A2, ACSL4, and GAPDH in indicated groups were determined by western blot assay. (B) Cell viability was assessed by CCK‐8 assay in indicated groups. (C) Fe^2+^, Fe^3+^, and total iron level in indicated groups. (D) Glutathione peroxidase activity levels were detected in indicated groups. (E) GSH contents and ratio of GSH/GSSG were measured in indicated groups. (F) The concentration of MDA was assessed by ELISA assay in indicated groups. **p < 0.01; ***p < 0.001; ****p < 0.0001.

We then overexpressed GPX4 in the HFLSs treated with Erastin. The efficacy of overexpression was confirmed in both mRNA and protein levels (Figure 6A, Figure S1). As expected, GPX4 overexpression notably rescued the role of Erastin in the expression of ADAMTS5, ACSL4 and SLC3A2 (Figure 6A). Consistent with previous results, Erastin notably inhibited the cell viability of HFLSs. However, GPX4 overexpression rescued the effect of Erastin and promoted cell survival of HFLSs (Figure 6B). The concentrations of different forms of iron were all notably lower in the GPX4 overexpression group compared to the Erastin‐treated group (Figure 6C). To evaluate the role of GPX4 overexpression in the antioxidant system and ferroptosis, we detected several markers in each group. Erastin treatment decreased glutathione peroxidase activity, GSH contents as well as GSH/GSSG ratio. GPX4 overexpression rescued its effect as well as increased the activity of the antioxidant system in HFLSs compared with the Erastin‐treated group (Figure 6D,E). Also, Erastin treatment increased the content of MDA, and GPX4 overexpression rescued its effect and reduced the level of MDA of HFLSs (Figure 6F). Here, we found that GPX4 overexpression promoted HFLSs survival through inhibiting ferroptosis.

### 3.6 GPX4 overexpression and Fer‐1 significantly ameliorate the pathological changes in the TMJOA rat model

**FIGURE 7:**
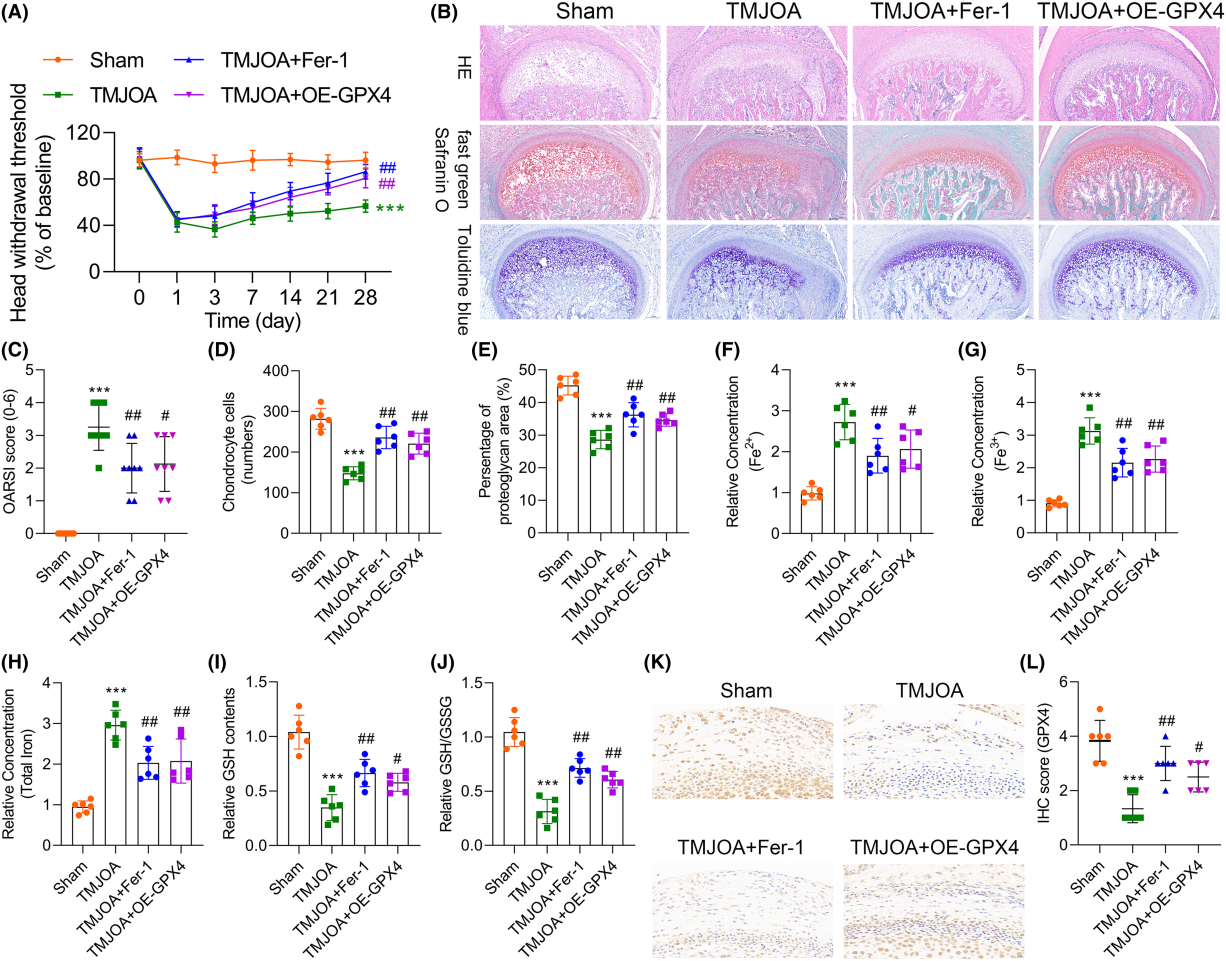
GPX4 Overexpression and Fer‐1 significantly improve the pathological changes in the TMJOA Rat Model. (A) The analgesic effect of celecoxib on chronic inflammation in rats was evaluated using the hot water tail‐flick test (HWT). (B) HE staining and Safranin O staining depicts the changes in cartilage structure and matrix proteoglycans of TMJ tissues. (C) Severity analysis of the TMJOA model using the OARSI scoring system. (D) Quantification of chondrocyte counts in the histological slices. (E) Statistical analysis of each group's percentage of proteoglycan area. (F–H) Assessment of iron concentration in various groups using an iron assay kit in TMJ synovial tissues. (I, J) The measurement of GSH contents and the ratio of GSH/GSSG in TMJ synovial tissues. (K, L) Immunohistochemistry (IHC) staining and evaluation of GPX4 expression intensity in TMJ tissues. Compared with Sham group, ***p < 0.001; Compared with TMJOA group, ##*p* < 0.01.

To confirm the impact of GPX4 and the iron death inhibitor in the TMJOA animal model, we first established a standardized TMJOA rat model, as depicted in Figure 7A. Chronic pain induced by MIA was utilized to induce TMJOA, and the loss of analgesic efficacy with celecoxib served as the standard for evaluating the TMJOA model. Subsequently, we dissected the temporomandibular joint tissues of each group of rats and assessed inflammatory cell infiltration (HE), the extent of cartilage damage (Fast Green/Safranin O), and the expression of proteoglycans (Toluidine Blue) (Figure 7B). Arthritis histopathological scores indicated that both Fer‐1 and GPX4 overexpression significantly reduced the severity of TMJOA (Figure 7C) and effectively prevented the loss of chondrocytes (Figure 7D). Compared to the TMJOA group, the levels of proteoglycans in the joint cavities of rats in the Fer‐1 and OE‐GPX4 groups significantly increased (Figure 7E), while iron content markedly decreased (Figure 7F–H). Antioxidant system activity was significantly enhanced in the Fer‐1 and OE‐GPX4 groups compared to the TMJOA group, where the antioxidant system activity was suppressed (Figure 7I,J). In the GPX4 IHC intensity score, we observed a significant reduction in GPX4 in the TMJOA model rats, whereas Fer‐1 and OE‐GPX4 rescued this reduction (Figure 7K,L). In summary, our data indicate that GPX4 expression is downregulated in TMJOA tissues in animal experiments, and iron death inhibitors significantly inhibit iron death while upregulating GPX4 expression. GPX4 overexpression suppresses iron death and promotes cell survival.

## 4 DISCUSSION

There are few effective therapeutic strategies for TMJOA due to the unclear pathology and mechanism for its progression. Patients with TMJOA would suffer from continuing pain and swelling difficulty which severely influenced life quality.^18^ Therefore, finding the key role of TMJOA progression and the characteristics of its pathology were critical for the clinic setting. Herein, we identified the dual role of ferroptosis in TMJOA progression and GPX4 was a key regulator for oxidative stress, ferroptosis, and cell survival of HLSs. Targeting GPX4 and ferroptosis may be an emerging area for TMJOA therapy.

Several targets and molecules were reported to regulate TMJOA progression previously.^19^, ^20^, ^21^, ^22^, ^23^ For instance, the HMGB1‐AKT axis mediated the vital role of glycyrrhizin in TMJOA progression.^24^ Glycyrrhizin prevented cartilage from degeneration by inhibiting inflammatory cytokines including HMGB1 and TLR4 which could effectively activate the AKT pathway in mice model.^25^ Also, exosomes isolated from mesenchymal stem cells prevented the progression of TMJOA by promoting s‐GAS synthesis, inhibiting the production of MMP13, which then inhibited inflammation and eventually maintained matrix homeostasis as well as joint repair.^26^ ALPK1, which was a regulator of chronic arthritis, also influenced TMJOA pathology and suppressed cartilage degradation by activating the NF‐κB signalling.^27^ Furthermore, the role of ALPK1 was also confirmed in the transgenic mice. ALPK1 knockout mice showed less damage to bone and cartilage.^27^ In Gli1^+^ osteogenic progenitors, the Hh signalling was proved to promote the TMJOA process by influencing the differentiation of osteogenic and maintaining bone homeostasis in the in vivo model.^28^ However, whether ferroptosis influences the process of TMJOA is still unclear. Our results indicated that ferroptosis played a dual role in TMJOA in vitro. Furthermore, targeting ferroptosis by inhibitors prevented HLSs from cell death. Here, we reported the important role of ferroptosis in TMJOA.

Ferroptosis was well known to be involved in several biological processes, especially cancers and inflammation‐related diseases.^10^, ^29^ Targeting ferroptosis has been well explored to develop cancer therapy strategy.^30^, ^31^ Ferroptosis mediated the effects of sorafenib in HCC.^32^ Furthermore, YAP signalling and ATF4 promoted sorafenib resistance through suppressing ferroptosis.^33^ HCAR1/MCT1 was a regulator of AMPK/SCD signalling by influencing the production of ATP and thus regulated the ferroptosis in HCC.^34^ In bone homeostasis, ferroptosis also damages bone marrow and FANCD2 was proven to prevent its effects.^35^ Researchers also observed that ferroptosis was notably activated in high fat‐induced bone loss.^36^ These results indicated that ferroptosis was a damager for bone homeostasis, which was consistent with our results that ferroptosis was activated in the TMJOA patients. Inhibiting ferroptosis notably maintains cell survival of HLSs.

GPX4 was a negative regulator of ferroptosis.^13^ Here, our results also indicated that GPX4 knockdown significantly promoted ferroptosis and GPX4 overexpression inhibited ferroptosis. GPX4 also positively regulated cell survival which was the opposite with ferroptosis. GPX4 was reported to be associated with osteosarcoma progression and bone marrow development.^37^, ^38^ Most studies proved that GPX4 exhibited dual functions through regulating ferroptosis,^39^, ^40^ which was consistent with our results. It is the first time to associated GPX4 with TMJOA progression.

This study investigated the impact of GPX4 (glutathione peroxidase 4) overexpression and Fer‐1 (ferroptosis inhibitor) on the TMJOA rat model. Results indicated that both Fer‐1 and GPX4 overexpression significantly reduced the severity of TMJOA and prevented chondrocyte loss. Furthermore, compared to the TMJOA group, the levels of intra‐articular proteoglycans significantly increased in the Fer‐1 and OE‐GPX4 groups, while iron content markedly decreased. This suggests that the Fer‐1 and GPX4 overexpression effectively suppress iron‐dependent cell death. The antioxidant system was significantly enhanced in the Fer‐1 and OE‐GPX4 groups, whereas it was inhibited in TMJOA tissues. This enhancement of antioxidant capacity may contribute to alleviating the pathological changes in TMJOA. Our research findings suggest that in the TMJOA animal model, the downregulation of GPX4 expression is associated with iron‐dependent cell death, while the iron‐dependent cell death inhibitor Fer‐1 and GPX4 overexpression significantly improve the pathological changes in TMJOA. These discoveries provide valuable insights for further investigations into therapeutic strategies for TMJOA, potentially leading to the development of novel treatment approaches to alleviate symptoms and pain in TMJOA patients. There are literatures showing the alleviation of TMJOA after secretome application.^41^, ^42^ In future studies, secretome can be further combined to further explore the role and mechanism of GPX4 in the TMJOA process.

## 5 CONCLUSIONS

Our study uncovered that GPX4 and ferroptosis could regulate the progression of TMJOA. GPX4 overexpression notably promoted cell survival of HLS by inhibiting ferroptosis. Inhibiting ferroptosis with ferroptosis inhibitors notably protected HLS cells. Targeting ferroptosis might be an effective therapeutic strategy for TMJOA patients in the clinic.

## AUTHOR CONTRIBUTIONS

**Chunyan Pang:** Formal analysis (equal); methodology (equal); validation (equal); writing – original draft (equal); writing – review and editing (equal). **Hongmei Zhang:** Investigation (equal); methodology (equal); visualization (equal); writing – review and editing (equal). **Yi Liu:** Formal analysis (equal); visualization (equal); writing – review and editing (equal). **Na Tang:** Validation (equal); writing – review and editing (equal). **Kun Tian:** Investigation (equal); validation (equal); writing – review and editing (equal). **Yandong Mu:** Data curation (equal); funding acquisition (equal); writing – review and editing (equal). **Xue Li:** Conceptualization (equal); data curation (equal); investigation (equal); project administration (equal); visualization (equal); writing – review and editing (equal). **Li Xiao:** Conceptualization (lead); data curation (equal); formal analysis (equal); funding acquisition (equal); project administration (equal); supervision (equal); writing – original draft (equal); writing – review and editing (equal).

## FUNDING INFORMATION

The research is supported by grants from the National Natural Science Foundation of China (82071168), the Key R&D project of the Science and Technology Foundation of Sichuan Province (2022YFS0290), Youth Talent Foundation of Sichuan Provincial People's Hospital (2018QN01), and Sichuan Provincial Cadre Health Research Project (Chuan Gan Yan 2019‐232, and Chuan Gan Yan 2022‐205).

## CONFLICT OF INTEREST STATEMENT

The authors declare that they have no competing interests.

## ETHICS STATEMENT

Approval of the research protocol by an institutional review board: The Ethics Committee of Sichuan Provincial People's Hospital approved this study [Lunshen (Yan) 2022‐233]. Informed consent: Patients' samples and information were collected under written informed consent. Animal studies: The animal experiment was permitted by the Institutional Animal Care and Use Committee of Sichuan Provincial People's Hospital.

## Supporting information


Appendix S1:

## Data Availability

The datasets used and analysed during the current study are available from the corresponding author upon reasonable request.
