# Supplementary figures and images for: Glutathione peroxidase 4 restrains temporomandibular joint osteoarthritis progression by inhibiting ferroptosis
Source: J Cell Mol Med. 2024 Apr 30;28(9):e18377. doi: 10.1111/jcmm.18377 (PMC11058612; doi:10.1111/jcmm.18377)

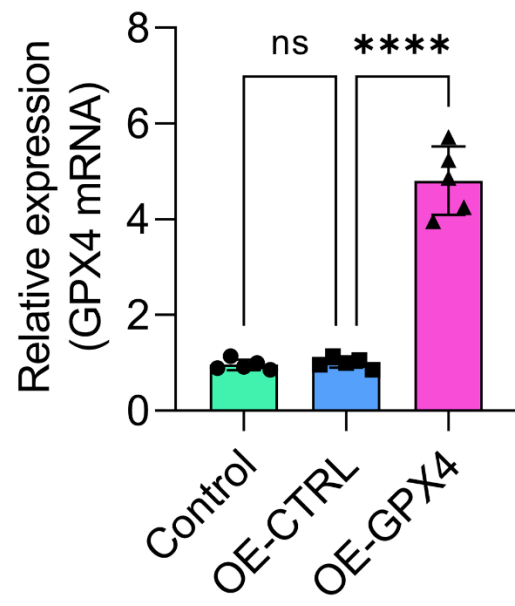

Figure S1. The mRNA level of GPX4 in indicated groups was determined by qRT-PCR assay.

Supplement: Supplementary file 1 — Appendix S1: [file JCMM-28-e18377-s001.pdf]
